# Dioxin induces Ahr-dependent robust DNA demethylation of the *Cyp1a1* promoter via Tdg in the mouse liver

**DOI:** 10.1038/srep34989

**Published:** 2016-10-07

**Authors:** Hesbon Z. Amenya, Chiharu Tohyama, Seiichiroh Ohsako

**Affiliations:** 1 Laboratory of Environmental Health Sciences, Center for Disease Biology and Integrative Medicine, Graduate School and Faculty of Medicine, The University of Tokyo, Tokyo, Japan; 2 Experimental Biology Laboratory, Faculty of Medicine, University of Tsukuba, Tsukuba 305-8575, Japan

## Abstract

The aryl hydrocarbon receptor (Ahr) is a highly conserved nuclear receptor that plays an important role in the manifestation of toxicity induced by polycyclic aromatic hydrocarbons. As a xenobiotic sensor, Ahr is involved in chemical biotransformation through activation of drug metabolizing enzymes. The activated Ahr cooperates with coactivator complexes to induce epigenetic modifications at target genes. Thus, it is conceivable that 2,3,7,8-tetrachlorodibenzo-*p*-dioxin (TCDD), a potent Ahr ligand, may elicit robust epigenetic changes *in vivo* at the Ahr target gene cytochrome P450 1a1 (*Cyp1a1*). A single dose of TCDD administered to adult mice induced Ahr-dependent CpG hypomethylation, changes in histone modifications, and thymine DNA glycosylase (Tdg) recruitment at the *Cyp1a1* promoter in the liver within 24 hrs. These epigenetic changes persisted until 40 days post-TCDD treatment and there was *Cyp1a1* mRNA hyperinduction upon repeat administration of TCDD at this time-point. Our demethylation assay using siRNA knockdown and an *in vitro* methylated plasmid showed that Ahr, Tdg, and the ten-eleven translocation methyldioxygenases Tet2 and Tet3 are required for the TCDD-induced DNA demethylation. These results provide novel evidence of Ahr-driven active DNA demethylation and epigenetic memory. The epigenetic alterations influence response to subsequent chemical exposure and imply an adaptive mechanism to xenobiotic stress.

The aryl hydrocarbon receptor (Ahr) is a highly conserved nuclear receptor that mediates toxic response to environmentally persistent organic pollutants (POPs) including polycyclic aromatic hydrocarbons (PAHs) and halogenated aromatic hydrocarbons (HAHs), such as 2,3,7,8-tetrachlorodibenzo-*p*-dioxin (TCDD)^1^. As a xenobiotic sensor, Ahr plays a role in chemical biotransformation through activation of drug metabolizing enzymes^2^. Ahr belongs to the basic helix-loop-helix (bHLH) superfamily of proteins that are involved in environmental response^3^. The ligand-activated Ahr dimerizes with its partner factor, Ahr nuclear translocator (Arnt), and binds xenobiotic responsive elements (XREs) found on target gene promoters, including phase I and phase II drug metabolizing enzymes^2^. *Cyp1a1* is the classical Ahr target gene that is induced by Ahr activation and is involved in the metabolism of PAHs to carcinogenic derivatives. Ahr also has critical roles in cardiovascular physiology^4^, immune regulation^5^, and hematopoiesis^6^.

DNA methylation and histone modifications are well characterized epigenetic marks that respond dynamically to diverse environmental factors^7^,^8^. As a regulator of multiple gene transcription networks, epigenetic modifications are anticipated to play a significant role in Ahr signalling. Indeed, the ligand-activated Ahr is known recruit several co-factors that have epigenetic modulatory functions, including the histone acetylase complexes (CBP/p300, p160 SRC1, NCoA-2 and p/CIP) and ATP dependent chromatin remodelers SWI/SNF^9^. The resultant changes in histone modifications have been described at the *Cyp1a1* enhancer and promoter regions^10^,^11^. Given the stability and dynamicity of epigenetic modifications, we hypothesized that Ahr activation may induce lasting epigenetic modifications and *Cyp1a1* transcription memory.

Enhanced drug metabolism is one of the major adaptive strategies during the development of pharmacokinetic xenobiotic tolerance^12^. Persistent epigenetic changes following initial drug metabolizing gene activation can ensure rapid and robust expression of drug metabolizing genes during subsequent drug exposures, resulting in enhanced deactivation of target chemicals^13^. DNA methylation can be altered in response to xenobiotic exposure^14^. Recent reports indicate that active DNA demethylation involves iterative oxidation of the methylated cytosine by the ten-eleven translocation proteins (Tet1, Tet2, Tet3) followed by recognition of the modified cytosine by thymine DNA glycosylase (Tdg), base excision repair (BER), and finally replacement with an unmodified cytosine^15^. There are a limited number of studies that describe coordinated targeting of the DNA demethylation proteins to specific genomic locations^16^. Nuclear receptors are likely candidates given their specificity in transcriptional regulation of target genes. Whether Ahr activation by TCDD induces DNA methylation alterations at the *Cyp1a1* promoter remains unclear. Furthermore, information on the potential role of epigenetic mechanisms in Ahr signalling is still limited.

This study provides novel evidence of Ahr-driven robust epigenetic modulation. The initial events involve Ahr-directed DNA demethylation and changes in histone modifications, followed by a long-term maintenance of this epigenetic configuration and *Cyp1a1* transcriptional memory. These epigenetic alterations influence response to subsequent chemical exposure and imply an adaptive mechanism to xenobiotic stress.

## Materials and Methods

### Reagents

Reagents used in this study were purchased from the manufacturers indicated in parentheses: 2,3,7,8-TCDD (purity >99.5%) (Cambridge Isotope Laboratory, Andover, MA, USA); DNeasy Blood & Tissue Kit, AllPrep DNA/RNA Mini Kit, RNeasy Mini Kit, QIAquick PCR Purification Kit and QIAquick Gel Extraction Kit (Qiagen, Hilden, Germany); PrimeScript RT reagent kit, TaKaRa Ex Taq and LA Taq (Takara Bio, Otsu, Japan); LightCycler 480 SYBR Green I Master (Roche Diagnostics GmbH, Mannheim, Germany); SimpleChIP Plus Enzymatic Chromatin IP Kit (Cell Signaling Technology, Danvers, MA, USA); Restriction enzymes HinP1I, HpaII, XbaI, Epimark 5 hmC and 5 mC Analysis Kit, HhaI and HpaII methyltransferases (New England Biolabs, MA, USA); MluI and XhoI (Toyobo, Osaka, Japan); Tissue-Tek O.C.T. Compound (Sakura Finetek, CA, USA); BrdU (Sigma-Aldrich, MO, USA); Hoechst 33342 (Dojindo, Japan); Hanks’ balanced salt solution (HBSS), Collagenase type IV, Lipofectamine2000, α-MEM, DMEM, fetal bovine serum (FBS), penicillin/streptomycin, and β-mercaptoethanol (Invitrogen, Carlsbad, CA, USA); pGL3-Basic Vector, phRL-TK Vector, and Dual-Luciferase Reporter Assay System (Promega, Madison, WI, USA); Site-Directed Mutagenesis Kit (Stratagene, CA, USA); HEPES, and Chemi-Lumi One reagents (Nacalai Tesque, Kyoto, Japan); porcine skin collagen (Nitta Gelatin Inc, Tokyo, Japan); siRNA oligos against mouse Ahr, Apex1, Tdg (Gm5806), and Non-targeting siRNA pool 1 (Dharmacon, PA, USA); siRNA against Tet1 (sc-154204), Tet2 (sc-154205), Tet3(sc-154206), and mouse monoclonal Ahr antibody A-2 (Santa Cruz Biotechnology, Santa Cruz, CA, USA), mouse monoclonal Anti-BrdU antibody (Becton Dickinson, CA, USA); Goat Anti-Mouse Alexa Fluor 488 (Abcam, MA, USA); Anti-Tet2 mouse monoclonal antibody and Immobilon-P PVDF membrane (Millipore, CA, USA).

### Animals

Adult C57BL/6J female mice aged 10 weeks were purchased from CLEA Japan (Tokyo, Japan). Ahr knockout (*Ahr*^−/−^) mice used in this experiment have been described previously^17^,^18^. Heterozygous (*Ahr*^*+/−*^) males and females were bred to obtain *Ahr*^−/−^ progeny. Animals were maintained on a 12/12 hour light/dark cycle, at a mean temperature of 23 ± 1 °C and 40% to 60% humidity. Laboratory rodent chow (Lab MR Stock; Nosan, Yokohama, Japan) and clean water were provided *ad libitum*. Animal experimentation protocols were reviewed and approved by the Animal Care and Use Committee, The University of Tokyo; and all experiments were performed in accordance with the relevant guidelines and regulations of the University of Tokyo.

### Treatment

Wild type female mice aged 10 weeks were orally treated with a single dose of TCDD (3 μg/kg bw) or vehicle (corn oil). This dose was selected on the basis that it does not cause overt toxic response in directly exposed mice and is approximately 60 times lower than the LD_50_ (182 μg/kg bw)^19^. Liver, kidney and brain samples were obtained at 6 hrs, 12 hrs, 24 hrs, 7 days, 20 days and 40 days post-TCDD treatment (*n* = 3). The tissues were snap-frozen in liquid nitrogen before being stored at −80 °C. Similarly, age-matched *Ahr*^−/−^ mice (*n* = 3) were given a single dose of TCDD (3 μg/kg bw) or corn oil. Liver samples were collected 24 hrs after TCDD treatment. Next, two groups of female mice (*n* = 3) were treated either with 3 μg/kg TCDD orally or corn oil. On day 40 after the initial dose, 100 ng/kg bw of TCDD was re-administered to both the TCDD pretreated group and the control group. Liver samples were collected 24 hrs after TCDD treatment for *Cyp1a1* expression analysis.

### DNA and RNA isolation

DNA was isolated from tissue and cell samples using the DNeasy Blood & Tissue Kit or AllPrep DNA/RNA Mini Kit. Total RNA was isolated using the RNeasy Mini Kit or AllPrep DNA/RNA Mini Kit. The purity and concentration of DNA and RNA were determined by spectrophotometry.

### RT-qPCR

The reverse transcription-quantitative PCR (RT-qPCR) technique used in this study has been previously described^20^. Briefly, RNA samples were first reverse-transcribed using the PrimeScript RT Reagent Kit. Subsequently, the cDNA was amplified using the LightCycler^®^ 480 SYBR Green I Master in a LightCycler^®^ 480 Instrument II. The primers that were used are listed in Supplementary Table S1. All oligonucleotides purified by gel-filtration were purchased from Hokkaido System Science (Sapporo, Japan). Absolute quantification of gene expression was performed using a standard curve derived from DNA amplicons prepared by the QIAquick Gel Extraction Kit followed by calculation of mRNA copy numbers per ng RNA^20^. The PCR was ran in duplicate and the average was used for quantification.

### Chromatin immunoprecipitation assay

The SimpleChIP^®^ Plus Enzymatic Chromatin IP Kit was utilized to perform chromatin immunoprecipitation (ChIP), as per the manufacturer’s protocol. Fresh liver tissue (50 mg) was crosslinked with 10% formaldehyde for 30 min. The chromatin fraction was digested with micrococcal nuclease and sonicated to give the desired fragment length (150 to 600 bp). The resulting protein-DNA complexes were then immunoprecipitated using the appropriate antibodies (Supplementary Table S2), followed by de-crosslinking. Next, qPCR was performed on the ChIP DNA (ChIP-qPCR) using the primer sets described in Supplementary Table S3. Relative amplicon levels were derived from a standard curve generated from serially diluted input DNA and fold enrichment was calculated relative to the control samples. The specificity of the assay was validated by normal rabbit IgG as the negative control.

### DNA methylation analysis

Locus specific quantification of methylation level was achieved using methylation-sensitive restriction enzyme-dependent quantitative PCR (MSRE-qPCR). Two methylation-sensitive restriction enzymes HinP1I and HpaII were used in this study. HinP1I targets the CpG sites −1039 and −500; while HpaII targets the −420 CpG upstream of the transcription start site (TSS) of the *Cyp1a1* gene. Briefly, an aliquot of genomic DNA was digested overnight (12 hrs) at 37 °C with either HinP1I or HpaII. Another DNA aliquot of similar concentration was digested with XbaI as a negative control since the target amplicon does not contain an XbaI site. The digested DNA was then subjected to qPCR using a primer set flanking the 3 CpGs of interest at the *Cyp1a1* promoter (see primer sets in Supplementary Table S4). Percentage of methylation level was calculated as the ratio of the copy number obtained from the HinP1I or HpaII digested DNA divided by the copy number obtained from the XbaI digested DNA.

### BrdU staining

Mice treated with 3 μg/kg TCDD or vehicle were administered with 50 mg/kg bw of BrdU intraperitoneally at 23 hrs post-TCDD treatment. One hour later, the animals were deeply anaesthetized with isoflurane and perfused with 4% paraformadehyde. The fixed livers and testes were cryoprotected in increasing concentrations of sucrose and then mounted in cryostat sectioning medium (Tissue-Tek^®^ O.C.T. Compound). 6 μm sections were stained with mouse monoclonal Anti-BrdU antibody and Goat Anti-Mouse Alexa Fluor^®^ 488. The sections were counterstained with Hoechst and visualised by fluorescence microscopy. Testes sections were used as the positive control where BrdU incorporating proliferating stem cells (spermatogonia) can be readily observed.

### 5 hydroxymethylcytosine analysis

Single CpG 5-hydroxymethylcytosine (5 hmC) level was analyzed using the Epimark 5 hmC and 5 mC Analysis Kit. Briefly, 10 μg of DNA was glucosylated using T4 Phage β-glucosyltransferase (T4-BGT) overnight, followed by simultaneous restriction enzyme digestion with MspI (sensitive to glucosylated-5 hmC), and HpaII (sensitive to the cytosine modifications 5 mC, 5 hmC, and glucosylated-5 hmC). The digested DNA was then analyzed by qPCR using primers flanking the −420 CpG site (Supplementary Table S4). The 5 hmC levels were calculated from the copy numbers using the following formula described by the manufacturer.


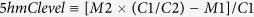


Where M2 is the qPCR copy number in the sample of genomic DNA treated with T4-BGT and digested with MspI, C1 is the copy number in the sample with genomic DNA only, C2 is the copy number in the sample of genomic DNA treated with T4-BGT only and M1 is the copy number in the sample of genomic DNA digested with MspI.

### Plasmid construction, point-mutation, and *in vitro* methyaltion

A *Cyp1a1* promoter fragment spanning −1534 bp upstream of the TSS through + 23 of exon1 was cloned from C57BL/6J mouse genomic DNA using LA Taq polymerase and gene-specific primers (Supplementary Table S5). The DNA amplicon was digested with MluI and XhoI, and then inserted into a pGL3-Basic luciferase vector to generate a pm*Cyp1a1*-Luc reporter vector.

In order to exclusively amplify the *Cyp1a1* promoter fragment from the plasmid DNA, but not the Hepa1c1c7 genomic DNA, we carried out site-directed mutagenesis of the pm*Cyp1a1*-Luc reporter vector targeting dinucleotides (TG to CA) at the 3′end of the forward primer using the Site-Directed Mutagenesis Kit. The mutation oligonucleotide sets are shown in Supplementary Table S6. The reporter plasmid pm*Cyp1a1*-Luc was methylated *in vitro* using HhaI and HpaII methyltransferases. HhaI methylates GCGC (HinP1I recognized-CpG site), while HpaII methylates CCGG (HpaII recognized-CpG site). Briefly, 5 μg of the plasmid was incubated for 5 hrs with the methyltransferase, supplemented with 32 mM S-adenosylmethionine. Successful methylation was verified by complete protection from respective restriction enzyme digestion.

### Cell culture and luciferase assay

The mouse hepatoma cell line, Hepa1c1c7, was cultured in α-MEM supplemented with 10% FBS, penicillin/streptomycin, and β-mercaptoethanol. Plasmid transfection was carried out using Lipofectamine2000. After co-transfection with the phRL-TK vector, luciferase gene reporter activity was measured by the Dual-Luciferase Reporter Assay System according to the manufacturer’s instructions.

### siRNA knockdown

The siRNA oligos for knockdown of Tet1, Tet2, Tet3, Ahr, Apex1, Tdg, and Non-targeting siRNA pool 1 were utilised. Non-targeting siRNA pool 1 (scramble) was used as the negative control. For knockdown experiments, the cells were plated on 24 well plates and 50 nM of the selected siRNA oligonucleotides were transfected with Lipofectamine2000. The transfection medium was changed 24 hrs post-transfection and target gene knockdown was verified after 48 hrs by RT-qPCR and western blot.

### *In vitro* demethylation assay

HhaI methyltransferase-treated pm*Cyp1a1*-Luc vector plasmid was co-transfected with siRNAs on day 0. The cells were then treated with 10 nM TCDD on day 1 and harvested 24 hrs post-treatment. DNA was isolated by the DNeasy Blood & Tissue Kit and plasmid methylation level was analyzed by MSRE-qPCR using primers targeting the mutated site (Supplementary Table S7). The cells used in this study were of the same origin and were treated on similar days.

### Primary hepatocyte isolation

Mouse primary hepatocytes were isolated by the two-step retrograde collagenase perfusion method described by Klaunig *et al*.^21^ Briefly, adult C57BL/6J mice were anaesthetized using isoflurane and the liver perfused with calcium and magnesium-free HBSS containing 5 mM glucose, 0.5 mM EGTA, and 25 mM HEPES through the inferior vena cava. Next, DMEM containing collagenase type IV at 100 CDU/ml, 5 mM glucose, 15 mM HEPES, and penicillin/streptomycin was used for liver digestion. The free hepatocytes were then filtered using a 100 μm nylon strainer and washed in DMEM containing 25 mM glucose, 10% FBS, 15 mM HEPES, and 100 nM dexamethasone. The cells were then plated on porcine skin collagen coated plates and cultured overnight in serum free low-glucose DMEM containing penicillin/streptomycin, 10 nM dexamethasone, and 5 mM HEPES. The hepatocytes were exposed to 10 nM TCDD dissolved in DMSO. Control cells were exposed to DMSO only. Samples were obtained at 0 hrs, 12 hrs, 18 hrs, 24 hrs, and 48 hrs post-TCDD exposure.

### Western blot

Hepa1c1c7 cells were quickly homogenized in 50 μl of ice-cold tissue sample buffer, sonicated, and then boiled for 5 min before storage at −80 °C. The cell lysate was separated on a 12% polyacrylamide and blotted onto the PVDF membrane. The membrane bound proteins were probed with the appropriate antibodies shown in Supplementary Table S7, mouse monoclonal Ahr antibody A-2, and anti-Tet2 antibody. The bands were visualized by the Chemi-Lumi-One chemiluminescence system.

### Immunocytochemistry

Cells were cultured in 24 well plates and fixed for 10 min using 4% paraformaldehyde in 0.2 M sucrose. The cells were then permeabilized with 0.1% TritonX-100 for 30 min. Nonspecific binding was blocked with 1% bovine serum albumin for 30 min before overnight incubation with mouse monoclonal Ahr antibody. Goat Anti-Mouse Alexa Fluor 488 was used as the secondary antibody. The cells were finally counterstained with Hoechst 33342.

### Statistics

All statistical analyses were done using StatView for Windows version 5.0 (SAS Institute, Cary, NC, USA). All results are represented as mean ± SE. Two-way analysis of variance (ANOVA) was used to analyze the effect of treatment in the MSRE-qPCR and 5 hmC data across all time points. The MSRE-qPCR data after siRNA knockdown was analyzed by one-way ANOVA followed by Fisher’s PLSD *post hoc* test. Student’s *t*-test was employed for the rest of the data. *P*-value less than 0.05 were considered statistically significant.

## Results

### Dioxin elicits *Cyp1a1* transcriptional memory

**Figure 1:**
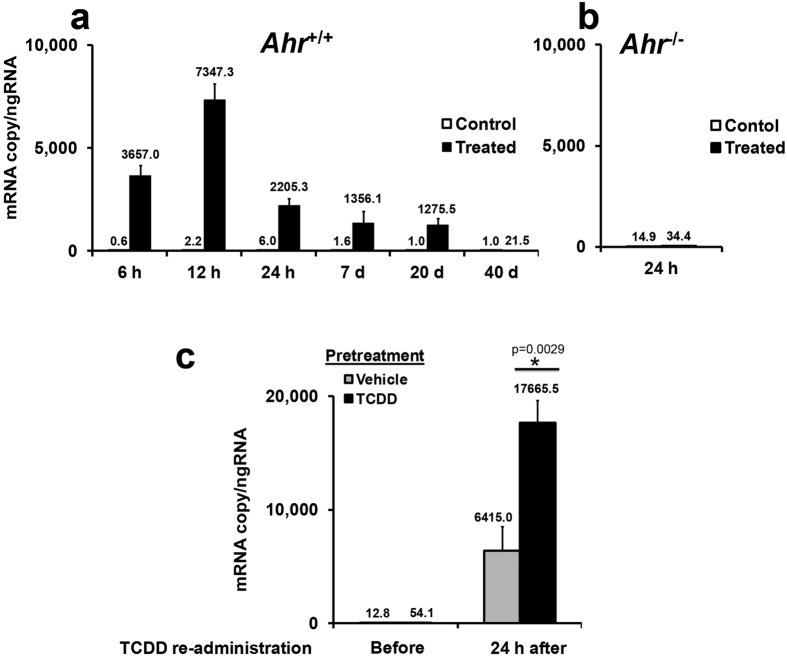
A single dose of TCDD induces *Cyp1a1* transcriptional memory. C57BL/6J female mice (wild type) were orally treated with TCDD at a single dose of 3 μg/kg bw, and livers were collected at indicated time points post-TCDD exposure. (**a**) Time-course changes of *Cyp1a1* mRNA expression levels in TCDD-treated animals (*n* = 3). (**b**) Lack of response in *Ahr*^−/−^animals. (**c**) Wild type female animals were pretreated with TCDD and repeat treated 40 days after the initial dose. Note an approximately three-fold super-induction of *Cyp1a1* in TCDD pretreated animals in comparison with non-pretreated animals (*n* = 3). *P < 0.05, Student’s *t*-test.

A single dose of TCDD induced *Cyp1a1* mRNA expression within 6 hrs which peaked at 12 hrs, and was followed by a gradual decrease after 24 hrs (Fig. 1a). Notably, at 40 days post-TCDD exposure, the level of *Cyp1a1* mRNA was very low (Fig. 1a). The *Ahr*^−/−^ animals did not respond to TCDD (Fig. 1b). To compare the inducibility of *Cyp1a1* transcription between control and TCDD-treated mice, we re-administered TCDD to the pretreated animals 40 days after the initial dose. There was an approximately three-fold higher induction of *Cyp1a1* in the TCDD-pretreated animals compared to non-treated control animals (Fig. 1c), indicating potential transcriptional memory.

### Dioxin induces stable changes in histone modifications at the *Cyp1a1* promoter

**Figure 2:**
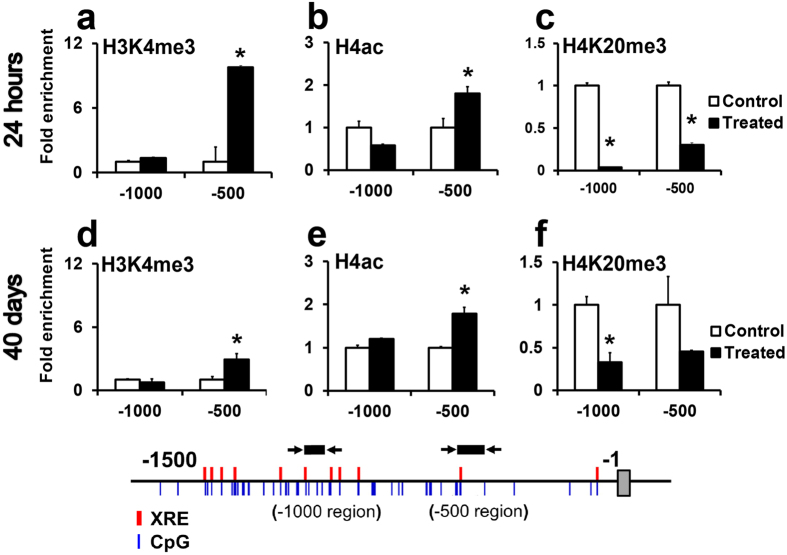
ChIP assay for histone modifications at the *Cyp1a1* promoter. C57BL/6J mice were treated with 3 μg/kg of TCDD and livers were collected at 24 hrs and 40 days post-TCDD treatment. (**a**–**c**) Levels of the histone modifications H3K4me3, H4ac and H4K20me3 as assayed by ChIP assay, 24 hrs after TCDD exposure, at the promoter regions indicated. (**d**–**f**) The histone modifications H3K4me3, H4ac, and H4K20me3 as assayed by ChIP assay, 40 days after TCDD exposure. Data are expressed as mean ± SE. (*n* = 3). *P < 0.05, Student’s *t*-test.

Histone modifications at the *Cyp1a1* promoter were analyzed 24 hrs and 40 days after TCDD treatment. At 24 hrs, the transcriptionally competent trimethylation of H3K4 (H3K4me3) and H4Ac were significantly increased while the repressive marker H4K20me3 was significantly decreased (Fig. 2a–c and Supplementary Fig. S1). Importantly, these epigenetic modifications were propagated to 40 days post-TCDD treatment (Fig. 2d–f). In particular, the increase in H3K4me3 and H4Ac at −500, and a decrease in H4K20me3 at the −1000 region were significantly enriched until 40 days post-TCDD exposure. On the other hand, *Ahr*^−/−^ mice did not show a treatment-related increase in H3K4me3 (Supplementary Fig. S2), indicating that this is an Ahr-dependent phenomenon. *β-actin* was used as the control gene (Supplementary Fig. S3). These stable changes in histone modifications, taken together with observed *Cyp1a1* hyperinduction indicate an Ahr-driven *Cyp1a1* epigenetic memory.

### Dioxin lowers methylation levels at the *Cyp1a1* promoter region

**Figure 3:**
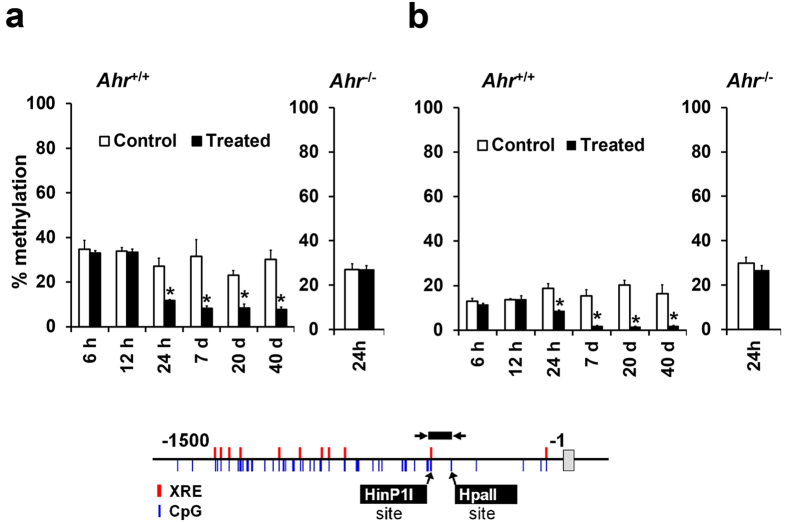
TCDD induces Ahr-dependent CpG demethylation of the *Cyp1a1* promoter. Animals were treated with 3 μg/kg TCDD and livers were sampled at the indicated time points. (**a,b**) The methylation level of the −500 CpG and −420 CpG, respectively, in the *Cyp1a1* promoter as measured by MSRE-qPCR. Data are expressed as mean ± SE. (*n* = 3). *P < 0.05, two-way ANOVA followed by Fisher's PLSD *post hoc* test.

To examine if Ahr activation elicits changes in the CpG methylation level on *Cyp1a1* gene promoter region, we measured DNA methylation levels in mouse liver samples after dioxin exposure. Treatment with TCDD induced the demethylation of two CpGs at the *Cyp1a1* proximal promoter (−500 and −420) within 24 hrs (Fig. 3a,b). The CpG at −1039 was entirely unmethylated (Supplementary Fig. S4). The DNA hypomethylation progressed steadily up until day 7 and the hypomethylated state was maintained to 40 days post-TCDD treatment (Fig. 3a,b). On the other hand, TCDD treated *Ahr*^−/−^ animals did not show lowered DNA methylation levels by 24 hrs, indicating that the TCDD-induced hypomethylation at the *Cyp1a1* promoter is an Ahr-dependent process (Fig. 3a,b). The relatively short duration taken for dioxin to induce DNA hypomethylation argued for involvement of an active demethylation mechanism. Passive DNA demethylation involves a cellular replication-dependent dilution of methylated cytosines in conditions where the DNA methylation maintenance methyltransferase is inhibited^22^,^23^. To clarify whether the demethylation involved passive removal of the methylated cytosine through cellular proliferation within 24 hrs, we performed BrdU staining on the TCDD treated livers. It was observed that TCDD does not induce cellular division in the mouse liver at 24 hrs post exposure (Supplementary Fig. S5). This suggests that the TCDD-induced hypomethylation is not a result of passive demethylation but is likely to result from active demethylation.

It is also worth noting that *Cyp1a1* transcriptional activation preceded *Cyp1a1* promoter demethylation. *Cyp1a1* mRNA was detectable by 6 hrs (Fig. 1a), earlier than CpG demethylation, which was observed at 24 hrs (Fig. 3a,b). Furthermore, *Cyp1a1* transcriptional extinction occurred by 40 days in the absence of CpG re-methylation, implying that CpG methylation loss possibly plays a role in epigenetic memory rather than in the induction of *Cyp1a1* gene transcription. The DNA demethylation was specific to the mouse liver, as the kidney and brain showed minimal or no methylation changes in response to TCDD exposure, respectively (Supplementary Fig. S6). Similarly, there was minimal *Cyp1a1* activation upon TCDD exposure in the kidney and brain (Supplementary Fig. S6).

### Ahr-driven active demethylation involves 5 hmC and the active DNA demethylation mediators Apex1, Tdg, and Tet3

Active DNA demethylation involves Tet protein-mediated enzymatic conversion of methylated cytosine (5 mC) to 5 hmC, 5-formylcytosine (5fC), and 5-carboxycytosine (5caC), followed by excision by Tdg and replacement with an unmethylated cytosine through the BER pathway^24^,^25^.

**Figure 4:**
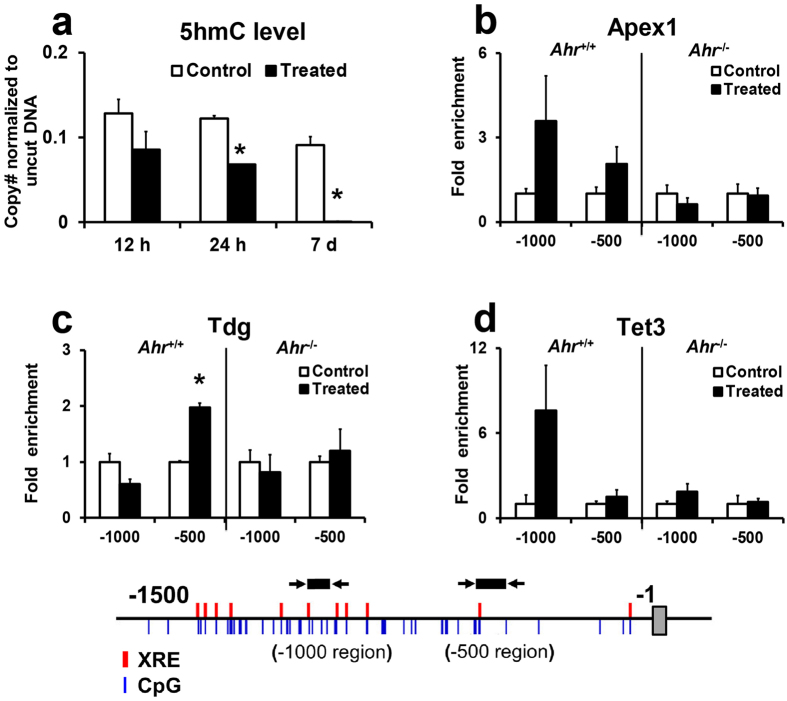
Ahr-driven active demethylation involves 5 hmC and active DNA demethylation mediators Apex1, Tdg, and Tet3. (**a**) 5-hydroxymethylcytosine levels after TCDD exposure. 5 hmC was analyzed by 5 hmC sensitive restriction enzyme digestion and qPCR. Data are expressed as mean ± SE. (*n* = 3). *P < 0.05, two-way ANOVA followed by Fisher’s PLSD *post hoc* test*. Cyp1a1* promoter occupancy of demethylation mediators as analyzed by ChIP assay in adult mouse livers; (**b**) Apex 1, (**c**) Tdg, (**d**) Tet3. Data are expressed as mean ± SE. (*n* = 3). *P < 0.05, Student’s *t*-test.

We show here that TCDD induced a gradual decline in 5 hmC content at the −420 CpG (Fig. 4a), a trend that is in agreement with the progressive CpG demethylation observed in Fig. 3b. ChIP assay for the demethylation factors Apex1, Tdg, and Tet3 revealed significant association of Tdg to the *Cyp1a1* proximal promoter (Fig. 4c). On the other hand, while there was an increase in Apex1 and Tet3 binding at the *Cyp1a1* promoter, it was found no to be statistically significant (Fig. 4b,d). This increase was especially noticeable at the −1000 region in TCDD treated *Ahr*^*+/+*^ mice but was absent in *Ahr*^−/−^ mice. Furthermore, TCDD did not alter the mRNA expression levels of Apex1, Tdg, Tet1, Tet2, and Tet3 (Supplementary Fig. S7). Tet1 had exceptionally an low mRNA copy number (Supplementary Fig. S7).

### Ahr, Tet2, Tet3, and Tdg are required for TCDD-induced DNA demethylation

**Figure 5:**
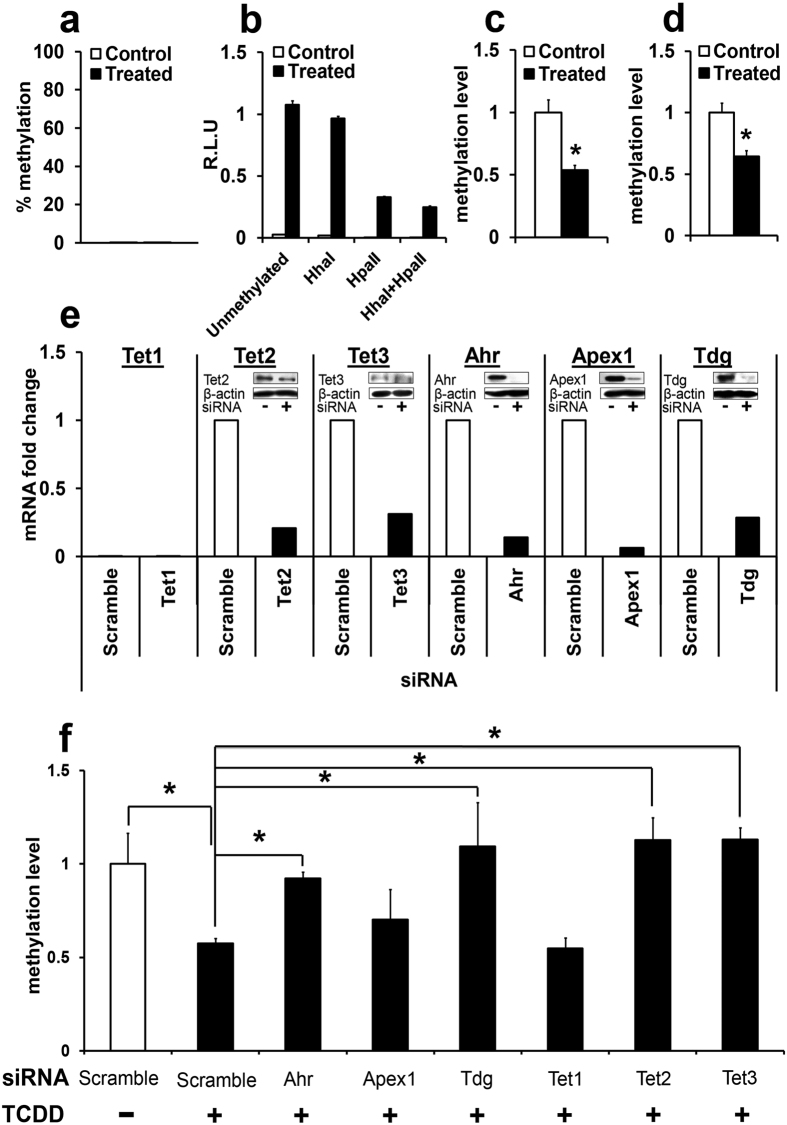
Ahr, Tdg, Tet2, and Tet3 are required for Ahr-directed DNA demethylation. (**a**) Hepa1c1c7 endogenous *Cyp1a1* promoter methylation levels at the target CpGs as analyzed by MSRE-qPCR. (**b**) The *in vitro* methylated *Cyp1a1* reporter plasmid response to TCDD as analyzed by luciferase expression. 100 ng of the methylated plasmid was transfected into Hepa1c1c7 cells. After treatment with 10 nM TCDD for 24 hrs, luciferase activity was measured by the Dual-Luciferase^®^ Reporter Assay System. (**c**,**d**) methylation levels of the −500 and −420 CpG sites respectively, in the *in vitro* methylated plasmids after TCDD exposure. Data are expressed as mean ± SE. (*n* = 3). *P < 0.05, Student’s *t*-test. (**e**) siRNA knockdown efficiency 48 hrs after transfection; mRNA levels were measured by RT-qPCR and protein levels by western blot. (*n* = 2). (**f**) Methylation level of the methylated plasmid after siRNA target knockdown and TCDD treatment. Data are expressed as mean ± SE. (*n* = 3). *P < 0.05, one-way ANOVA followed by Fisher's PLSD *post hoc* test.

To evaluate the functional involvement of DNA demethylation factors in Ahr-driven DNA demethylation, we utilized an artificially methylated plasmid (bearing a 1500 bp *Cyp1a1* promoter fragment) and siRNA knockdown against Ahr, Apex1, Tdg, Tet1, Tet2, and Tet3. Hepa1c1c7 was chosen as an optimal test cell line for the *in vitro* demethylation assay due to the low methylation levels in the endogenous *Cyp1a1* promoter (Fig. 5a). Other efforts to utilize primary hepatocytes for the functional study revealed that isolated *Ahr*^*+/+*^ hepatocytes have low *Cyp1a1* promoter methylation in comparison to the adult mouse liver and *Ahr*^−/−^ hepatocytes (Supplementary Fig. S8). Intranuclear Ahr localization in untreated hepatocytes was also observed (Supplementary Fig. S8). In contrast, Hepa1c1c7 cells showed nuclear Ahr localization only after TCDD treatment (Supplementary Fig. S8).

We found that methylation with HhaI methyltransferase (targeting HinP1I sites) did not change *Cyp1a1* reporter gene activation by TCDD. However, methylation with HpaII methyltransferase (targeting HpaII sites) or methylation with both HhaI and HpaII inhibited *Cyp1a1* reporter transactivation (Fig. 5b). Thus, HhaI methyltransferase was chosen for further experiments. Notably, 10 nM TCDD treatment of Hepa1c1c7 cells induced demethylation of the *in vitro* methylated plasmid within 24 hrs post exposure (Fig. 5c,d), replicating the *in vivo* findings (Fig. 3a,b). There was efficient target knockdown by 48 hrs post siRNA transfection (Fig. 5e).

Importantly, *in vitro* knockdown of Ahr, Tdg, Tet2, Tet3, and to a lesser extent Apex1, prevented TCDD-induced *Cyp1a1* promoter demethylation, suggesting that these genes are required for the Ahr-driven, TCDD-induced DNA demethylation (Fig. 5f). As expected, Tet1 did not ameliorate TCDD demethylation due to the low copy number (Supplementary Fig. S7).

## Discussion

Nuclear receptors are an attractive model for understanding the targeting of epigenetic modifier proteins to specific loci in the genome, given their wide roles in development, reproduction and homeostasis^26^. The present study provides novel evidence that Ahr drives robust epigenetic modulation at the *Cyp1a1* promoter. Ahr activation by dioxin led to Ahr-dependent changes in histone modifications (Fig. 2) and active DNA demethylation of the *Cyp1a1* proximal promoter in the adult mouse liver (Fig. 3). Maintenance of this hypomethylated state and open chromatin conformation contributed to *Cyp1a1* transcriptional memory.

The present findings suggest that *Cyp1a1* transcriptional memory may play a role in adaptive response to xenobiotic exposure and sheds light on the role of epigenetic memory in the development of xenobiotic tolerance. These findings are in agreement with a previous report, where the constitutive androstane receptor ligand TCPOBOP elicited epigenetic memory at the *Cyp2b10* promoter and increased zoxazolamine metabolism and hepatocyte *Cyp2b10* transcription memory^27^. Transcriptional memory of the *Cyp1a1* gene may imply increased detoxification of *Cyp1a1* substrates and possible activation of pro-carcinogens, such as benzo[a]pyrene. DNA hypomethylation after initial demethylation can remain for a long time after interleukin 2 activation in CD4 + cells^28^ or after demethylation of the *Tat* gene following glucocorticoid exposure in rat hepatoma cells^29^. In both cases, subsequent stimulation with antibody and dexamethasone, respectively, led to a faster and robust response, indicating that CpG demethylation acted as an epigenetic memory of initial exposure. Therefore, maintenance of histone modifications as well as DNA hypomethylation co-exist as epigenetic bookmarks for *Cyp1a1* hyperinduction in the liver of dioxin re-exposed mice.

In agreement with Ahr-driven DNA demethylation, nuclear receptor-directed active DNA demethylation has been observed at the *pS2* gene after estrogen receptor activation^30^, at the *Tat* promoter after glucocorticoid receptor activation^29^,^31^, and at adipocyte-specific genes by PPARγ-directed PARylation, Tet1 and Tet2 targeting^32^. The current model of active DNA demethylation involves enzymatic oxidation of 5 mC to 5 hmC, 5 fC, and finally 5 caC by the Tet proteins^24^,^33^. The latter two products are then recognized by Tdg and removed through base excision repair^25^,^34^. We observed a gradual decline in 5 mC and 5 hmC at the same CpG (−420) indicating that there was progressive demethylation that involved transition of 5 mC to the 5 hmC intermediate observed in Tet-mediated demethylation. A similar decline of 5 hmC has been observed in PPARγ-directed DNA demethylation in adipocytes^32^ as well as in DNA demethylation induced by hydroquinone^35^, phenobarbital^36^,^37^, and hydralazine^38^. In support of Tet protein involvement in Ahr-dependent active demethylation, we observed Ahr-dependent occurrence of Tet3 at the *Cyp1a1* promoter. Additionally, Tet2 and Tet3 knockdown suppressed dioxin-induced demethylation of an artificially methylated promoter. Tet1 has low expression levels in the adult liver^39^ and was thus not expected to contribute to the demethylation. ChIP assay of the BER proteins revealed that Apex1 and Tdg also occurred at the *Cyp1a1* promoter in an Ahr-directed manner, although only Tdg knockdown inhibited dioxin-induced demethylation of the methylated plasmid. Apex1 haploinsufficiency only results in a 35% decrease in BER in the mouse liver^40^, suggesting that another endonuclease such as Apex2 may compensate for low Apex1 protein levels.

Our analysis showed that the *Cyp1a1* promoter demethylation in the liver correlated with high *Cyp1a1* mRNA induction. However the brain and kidney, which had minimal DNA methylation changes after TCDD exposure, exhibited lower *Cyp1a1* expression. Differential tissue response to dioxin has been observed^41^, with the liver having considerably higher *Cyp1a1* response to dioxin than the brain^42^ and kidney^43^. The epigenetic configuration of the *Cyp1a1* promoter in various tissues may play an important role in tissue sensitivity and responsiveness. The role of differential CpG methylation in *Cyp1a1* inducibility has been demonstrated in various human cell lines^44^,^45^,^46^,^47^, as well as other species^48^.

Unexpectedly, the isolated hepatocytes used in the present study had low *Cyp1a1* promoter methylation in comparison to the adult mouse liver *in vivo*. Disruption of liver architecture during hepatocyte isolation has been shown to enhance cell cycle entry and binucleation (polyploidy) due to the oxidative stress resulting from liver digestion and cellular agitation^49^,^50^. Additionally, we observed intranuclear localization of Ahr in untreated hepatocytes. A similar picture has been observed in HepG2 cells being cultured under low glucose medium^51^, resulting in partial activation of *Cyp1a1*. The combined impact of increased hepatocyte binucleation and nuclear Ahr translocation on *Cyp1a1* promoter methylation may be of importance given that *Ahr*^−/−^ hepatocytes had higher methylation levels. In light of our findings at the *Cyp1a1* promoter and in consideration of the wider roles of Ahr in physiological function, we also anticipate the possibility of genome-wide epigenetic programming at Ahr binding elements and downstream target genes, possibly in a tissue- and cell-specific manner.

In conclusion, this study provides novel evidence of Ahr-dependent epigenetic regulation of target genes including active DNA demethylation. The Ahr-driven epigenetic memory influences response to subsequent chemical exposure and imply an adaptive mechanism to toxicological insult by xenobiotic chemicals.

## Additional Information

**How to cite this article**: Amenya, H. Z. *et al*. Dioxin induces Ahr-dependent robust DNA demethylation of the *Cyp1a1* promoter via Tdg in the mouse liver. *Sci. Rep*. **6**, 34989; doi: 10.1038/srep34989 (2016).

## Supplementary Material

Supplementary Information
