# Supplementary material for: Dioxin induces Ahr-dependent robust DNA demethylation of the Cyp1a1 promoter via Tdg in the mouse liver
Source: Sci Rep. 2016 Oct 7;6:34989. doi: 10.1038/srep34989 (PMC5054525; doi:10.1038/srep34989)
Supplement: Supplementary Information [file srep34989-s1.pdf]

Dioxin induces Ahr-dependent robust DNA demethylation of the *Cyp1a1* promoter via Tdg in the mouse liver

Hesbon Z. Amenya, Chiharu Tohyama, Seiichiroh Ohsako

Laboratory of Environmental Health Sciences, Center for Disease Biology and Integrative Medicine, Graduate School and Faculty of Medicine, The University of Tokyo, Tokyo, Japan.

*Corresponding author
ohsako@m.u-tokyo.ac.jp

Contents

Supplementary Table S1: Primer sequences for RT-qPCR……………………………………............. 2
Supplementary Table S2: Antibodies and antibody amounts used for ChIP assay……………….... 2
Supplementary Table S3: Primer sequences for ChIP-qPCR…………………….……………... 2
Supplementary Table S4: Primer sequences for MSRE-qPCR……………………………….. 2
Supplementary Table S5: Oligonucleotide sequences for mouse *Cyp1a1* promoter cloning........ 2
Supplementary Table S6: Oligonucleotide sequences for site-directed mutagenesis of mouse *Cyp1a1* promoter sequence……………………………….. 2
Supplementary Table S7: Primer sequences for plasmid methylation assay………….............. 2

Supplementary Figure S1: Histone modification changes on the *Cyp1a1* promoter after TCDD treatment……………………….……………………………... 3
Supplementary Figure S2: Dioxin treatment does not alter H3K4 trimethylation at the *Ahr*$^{-/-}$*Cyp1a1* promoter……………………………............. 4
Supplementary Figure S3: Histone modification changes on the *β-actin* promoter after TCDD treatment ……………………….. 5
Supplementary Figure S4: Liver DNA *Cyp1a1* methylation change in response to dioxin……... 6
Supplementary Figure S5: Dioxin does not induce proliferation in hepatic cells after treatment……………………………………………............. 7
Supplementary Figure S6: Differential tissue DNA methylation and *Cyp1a1* expression changes in response to dioxin…………………………... 8
Supplementary Figure S7: mRNA expression of known DNA demethylation mediators in response to dioxin treatment……………….................. 9
Supplementary Figure S8: *Cyp1a1* methylation and Ahr localization in primary hepatocytes and Hepa1c1c7 cells…………….................................10

Supplementary Table S1.Primer sequences for RT-qPCR (positions relative to ATG)

Gene	Forward	Reverse
mCyp1a1	(691)GTTACTGGCTCTGGATACCC(801)	(881)GACAATGCTCAATGAGGCTG(861)
mTdg	(925)CAACTGAAAGGCATTGAACG(944)	(1053)TGCCTCATAGCCTGGATCAT(1033)
mTet1	(4872)TGCTGGAGACTGTCGACTTG(4892)	(5060)GGACGTGGAGTTGTTCATCC(5040)
mTet2	(3427)ATCATGTTGTGGGACGGAAT(3447)	(3619)CATGCTCCAAGAACAACCAA(3599)
mTet3	(2768)ATTGTCAGAACGCCGTGATT(2788)	(2956)ACCGCAGGTGTTAGGGTCTT(2936)
mApobec1	(349)GCACGGCTTTATCACCACAC(369)	(536)CATACAGTTTCACCCACAGATGG(513)
mApex1	(138)CCCTCCAGATCAGAAAACCTC(159)	(334)AGGCAGCTCTTGCAGTTCAG(314)
mAhr	(1)ATGAGCAGCGGCGCCAA(17)	(171)TGCGGGAAGGGCAGCAG(154)

Supplementary Table S2: Antibodies and volume used ChIP assay

Antibody	Manufacturer	Volume used (ChIP)
Anti-Apex1 NB100-116	Novus Biologicals	4 µl
Anti-Tdg 13370-1-AP	Proteintech	10 µl
Anti-Tet3 C3	GeneTex	5 µl
Anti-H3K4me3	Cell Signaling Technology	4 µl
Anti-H4ac	Upstate Biotechnology	5 µl
Anti-H4K20me3	Upstate Biotechnology	5 µl

Supplementary Table S3.Primer sequences for ChIP-qPCR (positions relative to TSS)

Gene	Forward	Reverse
-100 region	(-164)TATCCGGTATGGCTTCTTGC(-141)	(+9)CACCTTCAGGGTTAGGGTGA(-11)
-500 region	(-534)TTCCTGTCCTGTGACCTCTG(-514)	(-358)TTGCACCCCTGAAACATTCA(-377)
-1000 region	(-1398)CAGGTTGAGTTAGACACGCCA(-1378)	(-1229)AGAGCAAAGGCCTGAACAGC(-1248)
β-actin promoter	(+162)GCAGTGAGGTACTAGCCACGA(+141)	(+9)GCTGTGGCGTCCTATAAAACC(-12)

Supplementary Table S4.Primer sequences for MSRE-qPCR (positions relative to TSS)

Gene	Forward	Reverse
-500 region	(-534)TTCCTGTCCTGTGACCTCTG(-514)	(-358)TTGCACCCCTGAAACATTCA(-377)
-1039 CpG	(-1137) CTCTTAAACCCCACCCCAAC(-1117)	(-1013)ACCCAGCTACCCAACTCACA(-1032)

Supplementary Table S5. Oligonucleotide sequences for mouse *Cyp1a1* promoter cloning (positions relative to TSS)

Forward	Reverse
(-1534)AAAAACGCGTTGTAACTAGAGTGGGAGG(-1517)	(+23)TTCTCGAGGCTCCAAGAACTACCACCTT(+3)

Supplementary Table S6. Oligonucleotide sequences for site-directed mutagenesis of mouse *Cyp1a1* promoter sequence (positions relative to TSS)

Sense	Antisense
(-531)CTGTCCTGTGACCTC**CA**GGCTGGGGTCGTTGC(-514)	(-531)GACAGGACACTGGAG**GT**CCGACCCCAGCAACG(-514)

Supplementary Table S7. Primer sequences for plasmid methylation assay (positions relative to TSS)

Gene	Forward	Reverse
m*Cyp1a1*m1	(-534)TTCCTGTCCTGTGACCTC**CA**(-514)	(-358)TTGCACCCCTGAAACATTCA(-377)

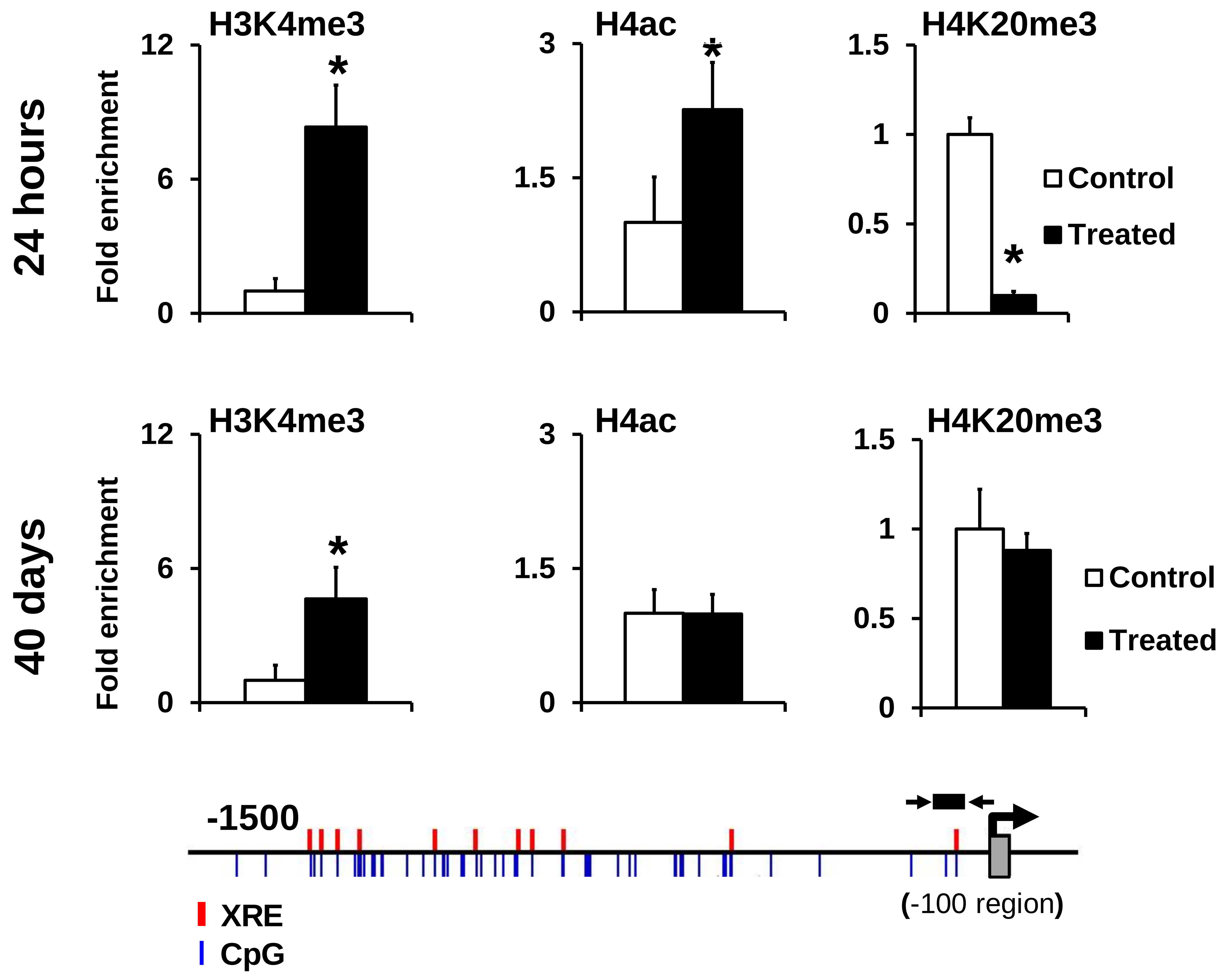

Supplementary Figure S1: Histone modification changes on the *Cyp1a1* promoter after TCDD treatment. Adult mice were treated with 3 μg/kg bodyweight and the level of H3K4me3, H4ac, and H4K20me3 were analyzed by ChIP assay, 24 hrs and 40 d after dioxin exposure. Data are expressed as mean ± S.E.M. (n= 3). *P<0.05, Student's t-test.

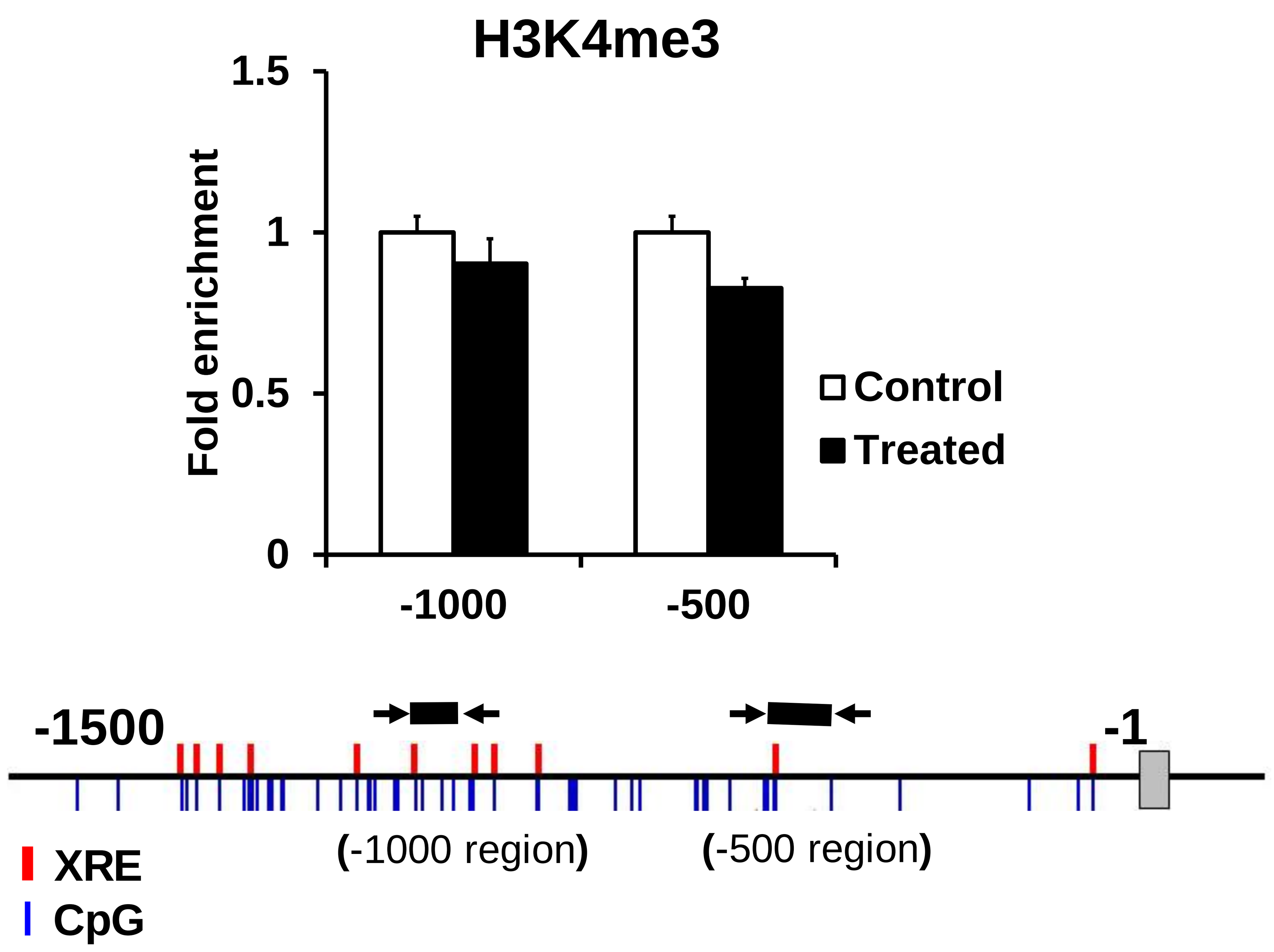

Supplementary Figure S2: Dioxin treatment does not alter H3K4 trimethylation at the *Ahr*$^{-/-}$ *Cyp1a1* promoter.Adult *Ahr*$^{-/-}$ mice were treated with 3 μg/kg bodyweight and the level of H3K4me3 was analyzed by ChIP assay, 24 hrs after dioxin exposure at the promoter regions indicated. Data are expressed as mean ± S.E.M. (*n*= 3).

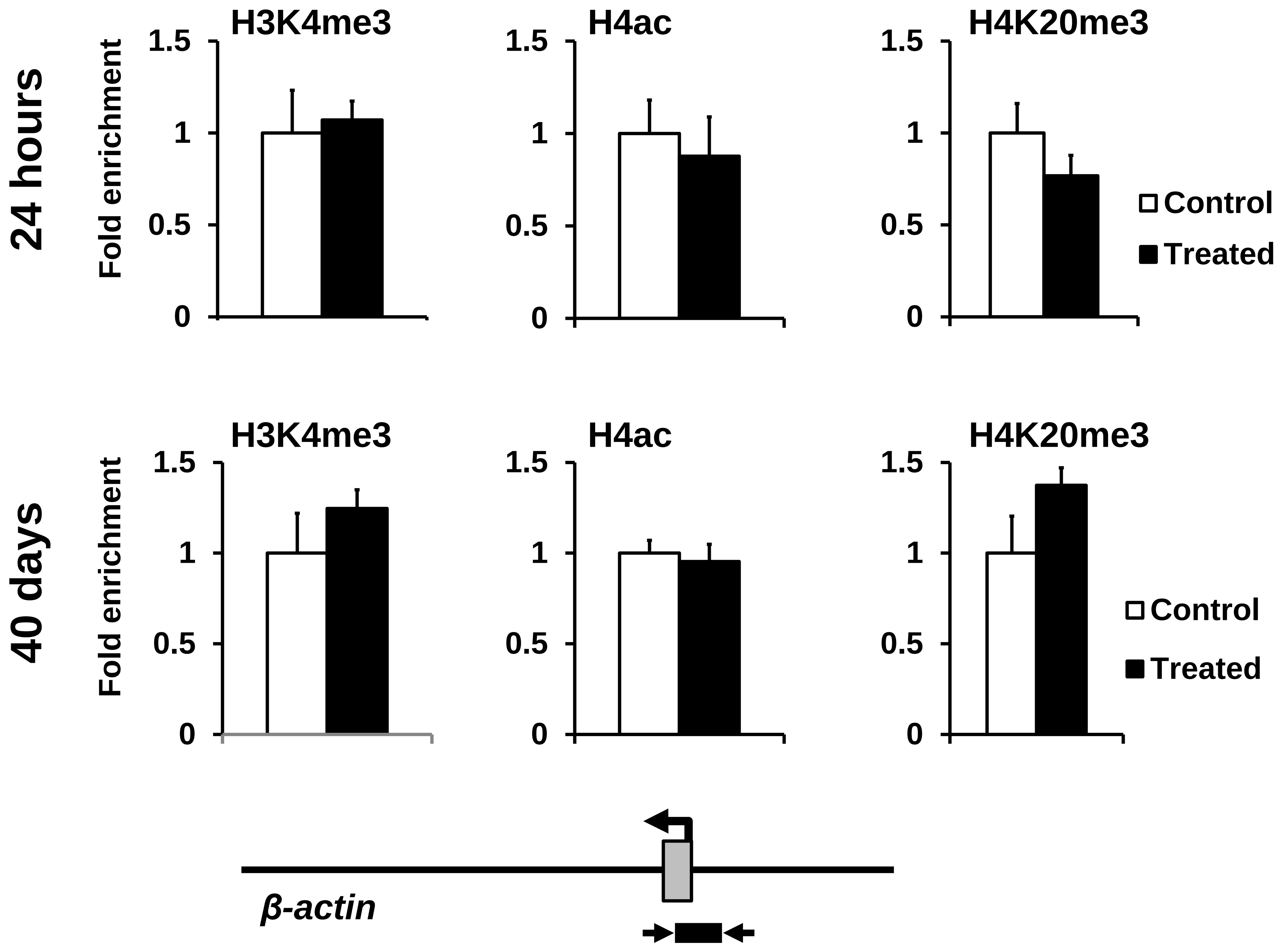

Supplementary Figure S3: Histone modification changes on the *β-actin* promoter after TCDD treatment. Adult mice were treated with 3 µg/kg bodyweight and the level of H3K4me3, H4ac, and H4K20me3 were analyzed by ChIP assay, 24 hrs and 40 d after dioxin exposure. Data are expressed as mean ± S.E.M. (*n*= 3).

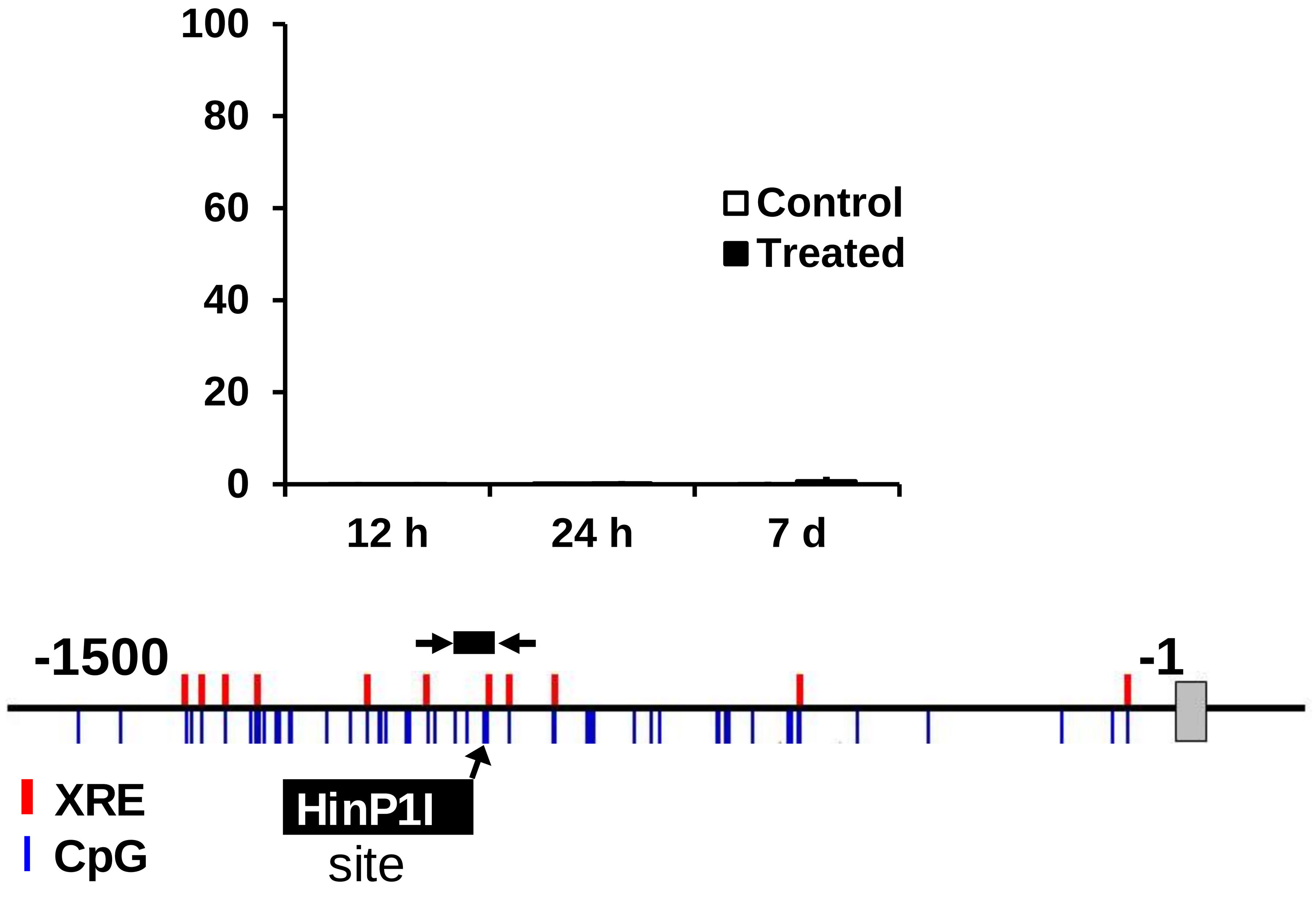

Supplementary Figure S4: Liver DNA *Cyp1a1* methylation change in response to dioxin. Adult mice were treated with 3 μg/kg bodyweight TCDD and the liver was sampled at shown time-points. The methylation level of the *Cyp1a1* promoter at -1039 CpG was measured by MSRE-qPCR. Data are expressed as mean ± SE (*n*= 3).

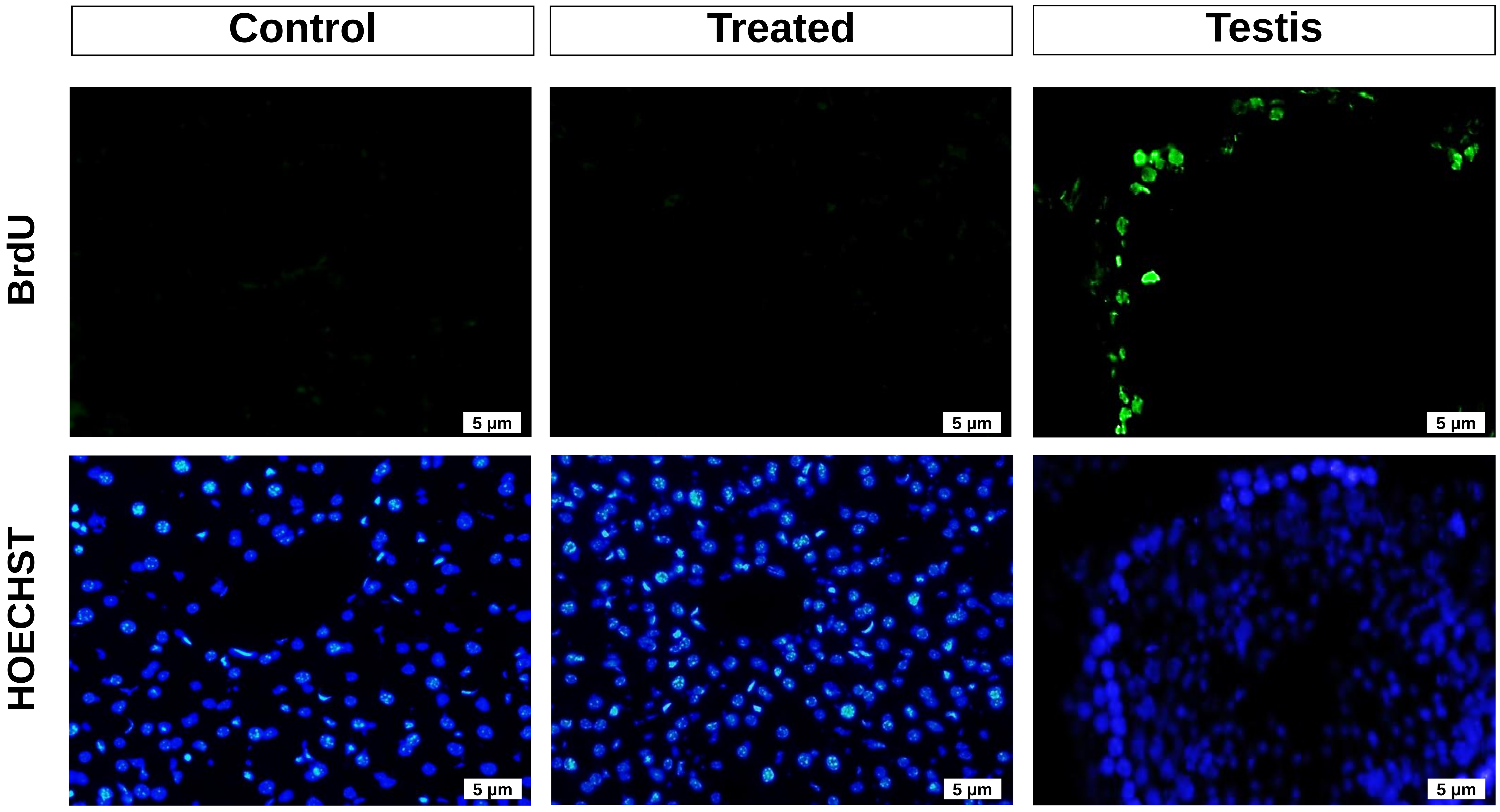

Supplementary Figure S5: Dioxin does not induce proliferation in hepatic cells after treatment. Representative photomicrographs of BrdU stained TCDD treated and control livers at 24 hrs post dioxin treatment.

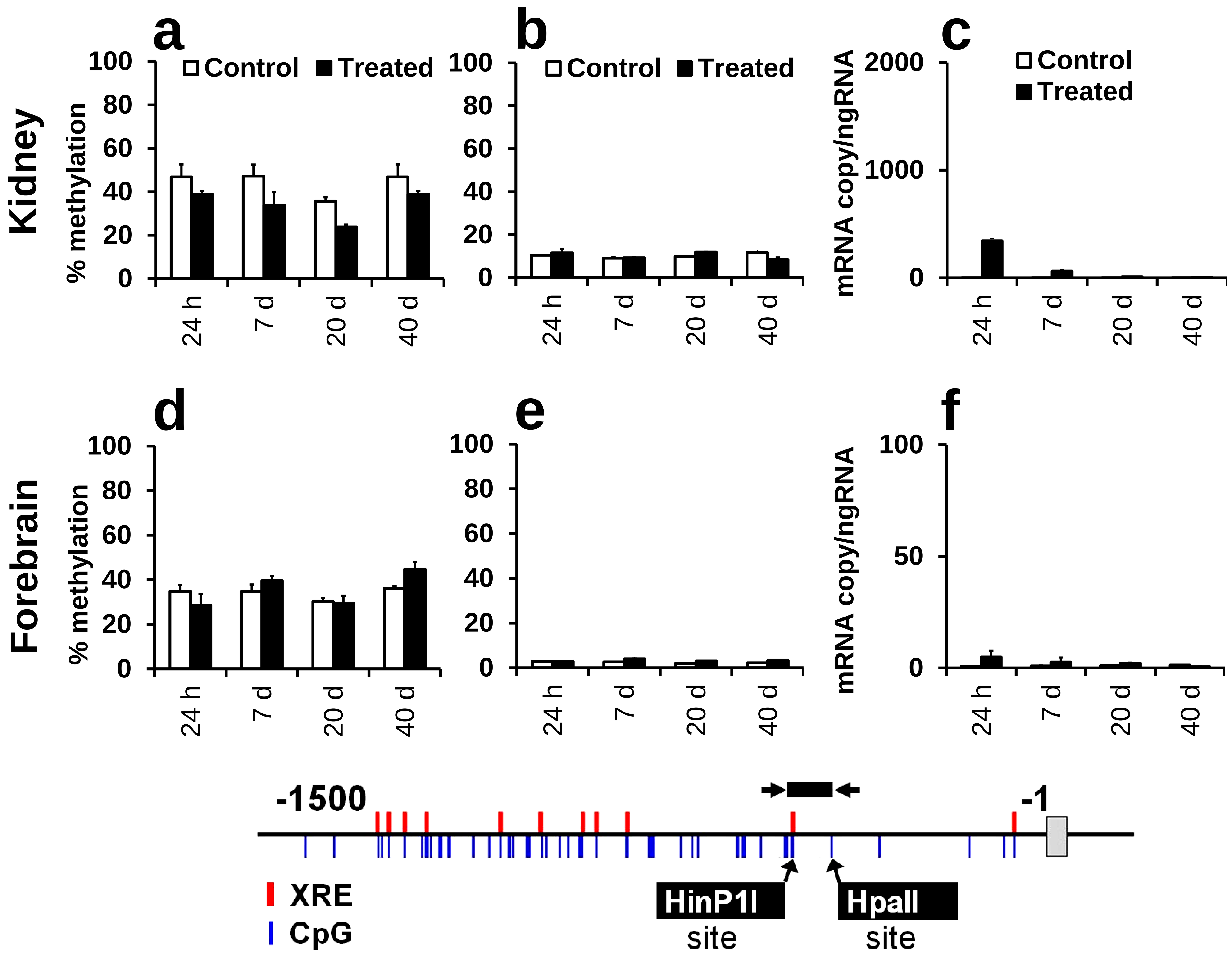

Supplementary Figure S6: Differential tissue DNA methylation and *Cyp1a1* expression changes in response to dioxin. Adult mice were treated with 3 μg/kg bodyweight TCDD and organs sampled at shown time-points. The methylation level of the mouse kidney and forebrain *Cyp1a1* promoter at -500 CpG (a,b) and -420 CpG (d,e), respectively, was measured by MSRE-qPCR. (c,f) time course *Cyp1a1* expression measured by RT-qPCR in the mouse kidney and forebrain. Data are expressed as mean ± SE (*n*= 3).

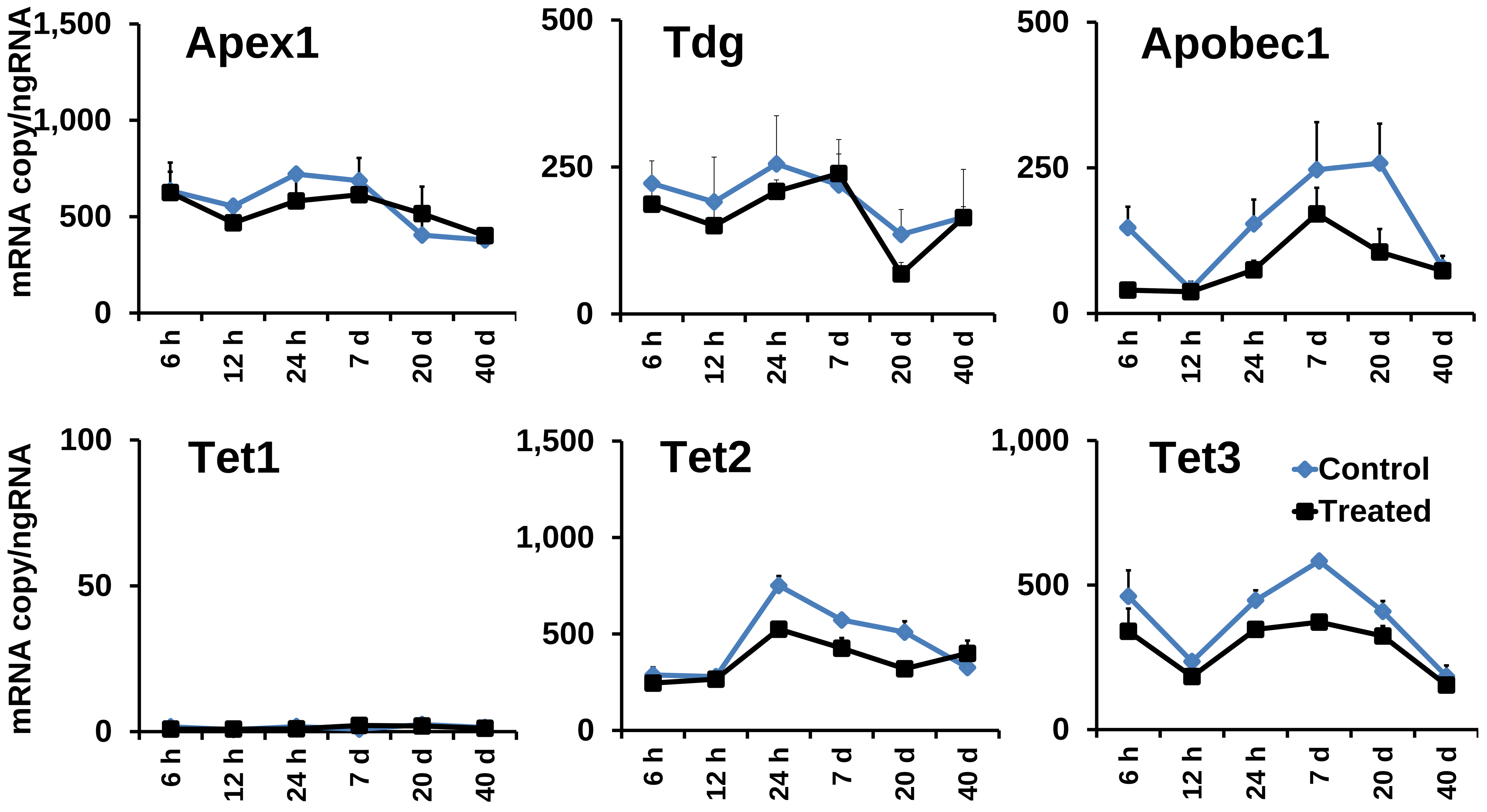

Supplementary Figure S7: mRNA expression of known DNA demethylation mediators in response to dioxin treatment. The expression level of each gene was measured by RT-qPCR in the adult liver at indicated time points after dioxin exposure. Data are expressed as mean ± SE. (*n*= 3).

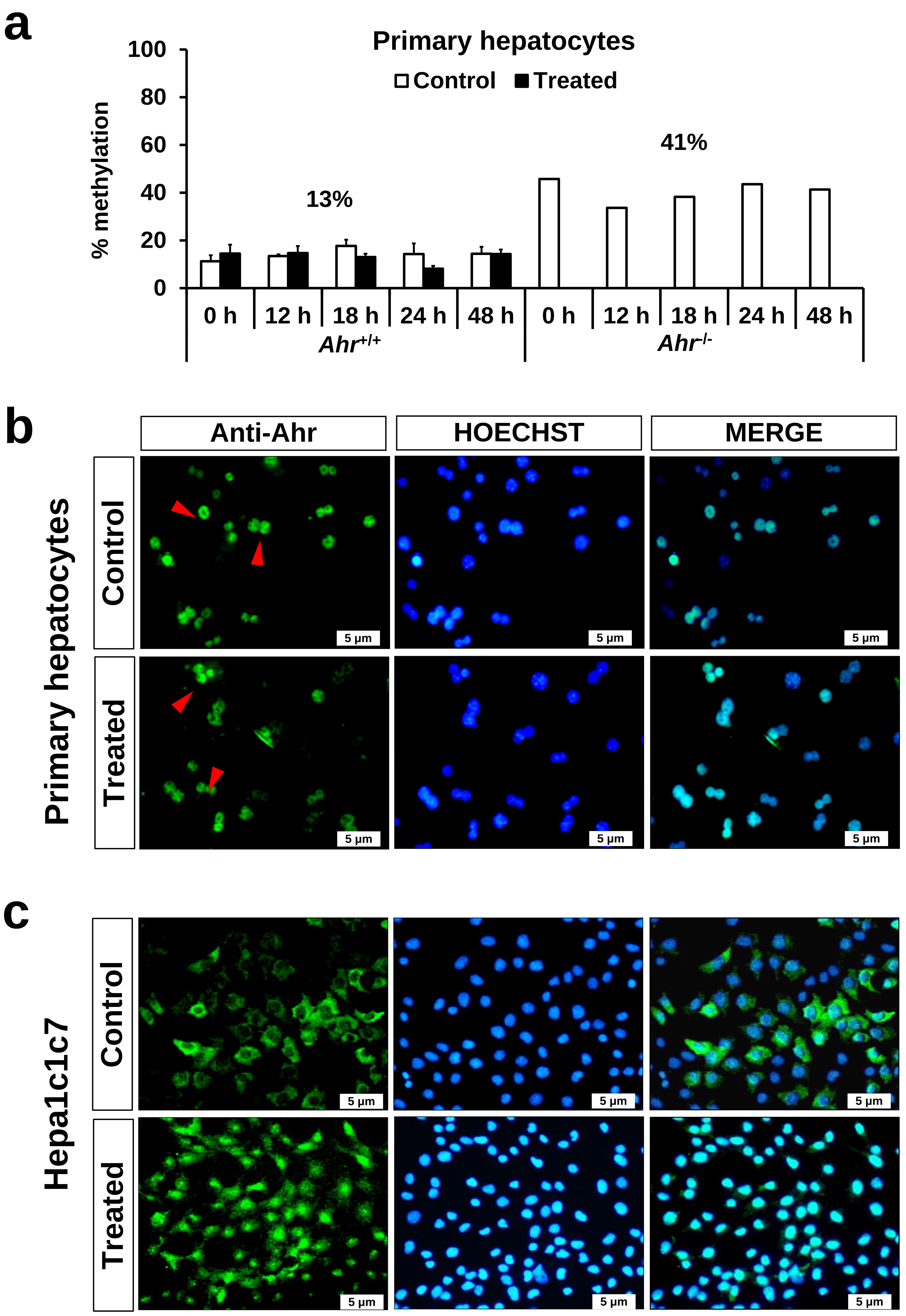

Supplementary Figure S8: *Cyp1a1* methylation and Ahr localization in primary hepatocytes and Hepa1c1c7 cells. (a) the methylation level of the *Cyp1a1* promoter (- 500 CpG) in *Ahr*$^{+/+}$ and *Ahr*$^{-/-}$ primary hepatocytes measured by MSRE-qPCR at various time points after isolation and after 10 nM dioxin exposure. Immunocytochemical staining of Ahr in; (a) primary hepatocytes and (b) Hepa1c1c7 cells at 24 hrs post dioxin exposure. Data are expressed as mean ± SE. (*n*= 3).
